# Determinants of self-rated health in an Irish deprived suburban population – a cross sectional face-to-face household survey

**DOI:** 10.1186/s12889-016-3442-x

**Published:** 2016-08-11

**Authors:** Catherine D. Darker, Erica Donnelly-Swift, Lucy Whiston, Fintan Moore, Joe M. Barry

**Affiliations:** 1 Public Health & Primary Care, Institute of Population Health Trinity College Dublin, Tallaght Cross, Dublin 24, D24 DH74 Ireland; 2 School of Dental Science, Dublin Dental University Hospital, Trinity College, Lincoln Place, Dublin 2, Ireland

**Keywords:** Self-rated health, Predictor, Community, Deprivation, Education, Employment, Chronic illness, Hospital use

## Abstract

**Background:**

Self-rated health (SRH) is amongst the most frequently assessed health perceptions in epidemiological research. While there is a growing understanding of the role of SRH, a paradigm model has yet to be widely accepted with recent studies concluding that further work is required in determining whether there are important predictors of SRH yet to be highlighted. The aim of this paper is to determine what health and non-health related factors were associated with SRH in a suburban deprived population in Dublin, Ireland.

**Methods:**

A cross sectional face-to-face household survey was conducted. Sampling consisted of random cluster sampling in 13 electoral divisions, with a sampling frame of 420 houses. Demographic information relating to the primary carer was collected. Health status of the primary carer was measured through SRH. Household level data included the presence or absence of persons with a chronic disease, persons who smoked, persons with a disability and healthcare utilisation of general practitioner and hospital level services. A logistic regression model was utilised in the analysis whereby the odds of primary carers with poor SRH were compared to the odds of carers with good SRH taking health and non-health related factors into account.

**Results:**

Of the 420 households invited to participate a total of 343 were interviewed (81.6 % response rate). Nearly half of the primary carers indicated their health as being ‘good’ (*n* = 158/342; 46.2 %).

Adjusting for the effects of other factors, the odds of primary carers with second level education were increased for having poor SRH in comparison to the odds of those with third level education (OR 3.96, 95 % CI (1.44, 11.63)). The odds of primary carers who were renting from the Council were increased for having poor SRH compared to the odds for those who owned their own property (OR 3.09, 95 % CI (1.31, 7.62)). The odds of primary carers that were unemployed (OR 3.91, 95 % CI 1.56, 10.25)) or retired, ill or unable to work (OR 4.06, 95 % CI (1.49, 11.61)) were higher for having poor SRH than the odds of those in employment. If any resident of the household had a chronic illness then the odds of the primary carer were increased for having poor SRH compared to the odds for a primary carer in a household where no resident had a chronic illness (OR 4.78, 95 % CI (2.09, 11.64)). If any resident of the household used the local hospital, the odds of the primary carer were increased for having poor SRH compared to the odds for the primary carer in a household where no resident used the local hospital (OR 2.01, 95 % CI (1.00, 4.14)).

**Conclusions:**

SRH is affected by both health and non-health related factors. SRH is an easy to administer question that can identify vulnerable people who are at risk of poor health.

**Electronic supplementary material:**

The online version of this article (doi:10.1186/s12889-016-3442-x) contains supplementary material, which is available to authorized users.

## Background

Self-rating of health (SRH), based on a simple question such as “How would you rate your health?” is one of the most frequently employed health measures in large scale general population research [1]. It has been used to examine the relationship between health and a wide range of social and economic factors, including educational attainment [2–5], occupational status [4, 5], deprivation [6, 7], social capital [8–10], age and gender [11]. Despite its brevity SRH is a multifaceted paradigm that is informed by an individual’s cognitive awareness and their experience of their personal physiological, psychological and sociological health [12–14]. In Ireland SRH was included in the national census data for the first time in 2011 [15], with approximately 88 % of the population rating their health as ‘very good’ or ‘good’, 8 % as ‘fair’ and 2 % rating their health as either ‘bad or very bad’ [16]. This is considerably higher than the Organisation for Economic Cooperation and Development (OECD) average of just 69 % of the adult population (aged over 15) rating their health as ‘good’ or ‘very good’ [17].

The social gradient in health, disease and mortality is one of the most widely observed and consistent findings in international epidemiological research [18–20]. A recent study in Australia demonstrated the need to consider deprivation levels within communities in relation to SRH [6]. Neighbourhood deprivation (both physical and social) is associated with lower collective efficacy, trust, and social capital, and higher levels of social and physical disorder, fear of crime, and racism. This demonstrates the relationship between neighbourhood deprivation and SRH is mediated by the social and physical characteristics of the area [21]. This emphasises the importance of studying the multilevel structure of disadvantage, and indeed whether there is a double disadvantage, when considering SRH.

A number of studies have shown SRH to be a strong, consistent predictor of morbidity and mortality across varied populations, even after adjustment for confounding factors such as age, sex, and prior clinical history [6, 13, 14, 22–25]. Individuals with poor SRH have been found to have between two and five times higher risk of death compared with individuals who reported good SRH after two to twenty eight years of follow up [13, 14, 23–25]. SRH has been found to be a significant predictor of onset of chronic disease among US adults aged over 50 years including cardiovascular diseases (CVD), arthritis, diabetes, lung disease and stroke [26]. The relationship between SRH and other health outcomes including chronic disease incidence, diabetes complications, physical and cognitive functional limitations, health services use, and clinical biomarkers have also been investigated [27–31]. A high SRH was found to be associated with lower risk of vascular events and major complications in type 2 diabetics in a longitudinal cohort study of over 7000 patients [31]. Living with a person with a chronic illness has also been shown to be associated with lower SRH [26, 31, 32].

SRH is an active cognitive process, consisting of several stages, that is not guided by formal, agreed rules or definitions of health but lies at the cross roads of culture and biology. A unified conceptual model of SRH outlines the complicated process, which determines a person self-assessment of their own health status. This involves a determination of the cultural and historical varying concepts of ‘health’; personal reference groups that the individual draws from, early health experiences, health expectations, positive or negative dispositions and cultural conventions [33]. The perception of health status is not a static phenomenon but rather one that changes with new ‘information’. Human judgements are based on multiple psychological processes, which are subject to internal and external influences [34]. With this in mind, the theoretical framework of the International Classification of Functioning, Disability and Health (ICF) [35] was used to create a better understanding of factors associated with SRH [36]. This demonstrated that SRH had an independent association with five variables representing ICF body functions, activities and personal factors.

Nonetheless a complex relationship exists between SRH and health-related behaviours. Health-related behaviours included in SRH studies often include smoking status, dietary assessments, physical activity, body mass index (BMI) or presence of obesity, and alcohol consumption [32]. Physical activity has been shown to have a relationship with SRH in a number of studies with associations between higher levels of physical activity and better SRH [37] and a lack of regular physical activity associated with poorer SRH [38]. The experience of stress has been positively associated with poor SRH [39] with women in particular reporting higher stress than their male counterparts [39, 40]. Conflicting findings have emerged concerning the relationship between SRH and smoking, alcohol consumption, and dietary behaviours [30, 41–46]. Health behaviours have been shown to mediate the relationship between SRH and mortality, and this effect often differs by gender and/or duration of effect [29, 47, 48]. Layes et al. [49] determined that individuals who engaged in healthy lifestyles were actually more likely to be pessimistic regarding their health status.

Understanding how health and non-health factors affect health through their relationship with SRH is important in developing effective health policies and for future health planning. A paradigm model of the factors which influence SRH has yet to be fully understood and accepted, with further research necessary [1, 50].

## Methods

### Aim

The aim of this paper is to answer the following research question - what health and non-health related factors predict SRH in a suburban deprived population in Dublin, Ireland? This maps onto the study hypothesis, that SRH will be associated with both health and non-health related factors.

### Design

A cross sectional face-to-face household survey of the health assets and needs assessment (HANA) relating to the health and wellbeing of a suburban deprived community of South Dublin, Ireland.

### Setting and sampling

The sampling process closely followed a health needs assessment study conducted in the same geographical area in 2001 [51, 52]. Deprivation is a state of “observable and demonstrable disadvantage relative to the local community to which an individual belongs” [53]. Deprivation has a role to play in terms of health status [6, 7] and service uptake [54] and therefore it was necessary to take account of this factor when selecting the sample. The Small Area Health Research Unit (SAHRU) of the Department of Public Health & Primary Care at Trinity College Dublin has developed a deprivation index (DI) for health and health services research. The most recent SAHRU DI, based upon national census data for 2011, utilises four indicators to determine the classification of deprivation [55]; unemployment, low social class, no car and government funded housing. The DI score ranges from one to ten, where one is least deprived and ten is most deprived.

**Fig. 1:**
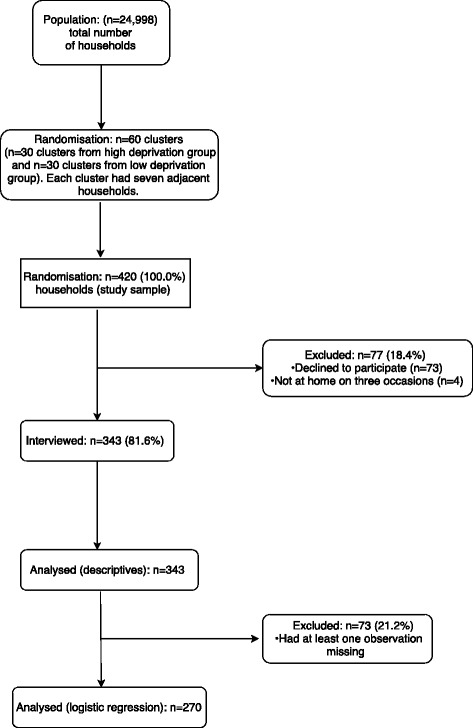
Flow diagram of study participants

Sampling consisted of random cluster sampling in 13 electoral divisions (EDs) in a suburban deprived area of South Dublin. There were a total of *n* = 24,998 households in the EDs. An ED is the smallest legally defined administrative area in Ireland for which Small Area Population Statistics are published from the national census [15]. The households were partitioned into clusters. A SAHRU DI score was applied to each cluster. A systematic sample of clusters was selected consisting of 30 clusters from a high deprivation group of EDs and 30 clusters from a low deprivation group of EDs with each cluster consisting of seven adjacent houses. This yielded a total sampling frame of 420 households Fig. 1.

### Procedure

The study was advertised in the local area and general practitioners were informed of the study through an information letter. Letters were sent to 420 randomly selected households, inviting the primary carers to participate in the survey. The primary carer was identified as the person in the household who manages the welfare and health of the family/household. In a house of renters this was the person who pays the bills or whose name was on the rent agreement. Interviewers were rigorously trained in the survey methodology. Three members of the research team (CD, JB & LW) conducted a half-day training session. This consisted of the background, context and purpose of the study, the questionnaire, informed consent and the working alone procedures. Role-plays were employed to familiarise the data collectors with the questionnaire and surrounding procedures. The quality of data collection was assessed when the first batch of questionnaires were returned. Questionnaires were assessed for any errors and issues identified were discussed with the data collectors. Data collectors were also offered an opportunity to ask questions or clarify any concerns they had relating to data collection procedures or the questionnaire. Each interviewer elicited informed written consent from each primary carer before the interview commenced.

### Measures

The questionnaire consisted of a range of different topics; demographic information relating to the primary carer such as employment status, house occupancy status, and level of health cover. Some questions were at the level of the individual primary carer level, while other questions were at the household level. Depending on the number of residents in each household questionnaires took 20 to 30 min to complete.

### Health status

Health status of the primary carer, was measured through administration of a range of validated instruments measuring SRH, social capital, physical activity and stress experienced, including symptoms resulting from stress experienced.

### Self reported health (SRH)

SRH was assessed by the answer to a single item ‘How is your health in general?’. There were five response categories: ‘very good, ‘good’, ‘fair’, ‘bad’, and ‘very bad’. This question has become a standard measure for SRH and due to its format can be compared with Irish and international data [15]. This question has been employed in the national Census of Ireland in 2011 and 2016. For analysis this variable was collapsed into ‘good self-rating of health’ consisting of ‘very good’ and ‘good’. ‘Fair’, ‘bad’ and ‘very bad’ were collapsed to make up ‘poor self-rating of health’. The collapse of answer categories was determined by those who answered ‘fair’ having poorer health than those who answered ‘good’ or ‘very good’.

### Physical activity

Four questions assessed primary carers’ levels of physical activity. The number of times severe, moderate and mild physical activity was engaged in for more than 20 min during an average week was recorded along with the number of days in an average week that the primary carer walked for more than 30 min. These questions asked in the 2002 Survey of Lifestyle, Attitudes and Nutrition survey (SLAN) were adapted from the International Physical Activity Questionnaire (IPAQ) [56]. This enabled the employment of the IPAQ scoring protocol to assign primary carers to categories based on levels of physical activity. The IPAQ has been shown to be a valid and reliable measure of physical activity with repeatable data and criterion validity with a median of 0.30 [56, 57].

### Level of healthcare cover

In Ireland there is a two-tier health service; state provided general medical (primary care) services (known as GMS ‘medical card’) to those households on low income (just over 40 % of the population [58]) and the remainder who may either be privately insured or pay out-of-pocket for their healthcare.

### Stress

Stress was assessed by answers to a number of items used in a previous study [51]. Firstly, respondents were asked “Have you experienced stress within the last 12 months?”. If this item was answered positively then a number of questions gathered further information about the stress, such as reason for stress, scale of the problem, symptoms experienced due to stress and steps taken as a result. These questions have previously been employed with a similar population through a previous cross sectional study conducted within the same suburban deprived area in 2001 [51].

Household level data included demographic information for all people living in the house, the presence or absence of a person living in the household with a chronic disease, whether the household contained a person who smoked, whether any member of the household was in receipt of a disability benefit and healthcare utilisation of general practitioner and hospital level services. (See Additional file 1 for copy of survey instrument).

### Analyses

This study adopts a logistic regression model, to examine the odds of primary carers with poor SRH compared to the odds of carers with good SRH, taking health and non-related health factors into account.

Fixed effects classified in the model were demographic characteristics, house occupancy status, level of health cover and health status characteristics. A random intercept was included in the model to account for cluster variation. Information criterion and likelihood ratio tests were used to evaluate goodness of fit. Akaike’s information criterion (AIC) considers both error and the principle of parsimony. AIC penalises a model for too many parameters. Bayesian information criterion (BIC) is an alternative to AIC. BIC takes sample size into consideration and is slower to be drawn toward more complex models as sample size increases [59]. Receiver operating characteristic (ROC) curve and area under the curve (AUC) were used to examine sensitivity and specificity. The ROC curve is a graphical plot of sensitivity versus 1-specificity. If perfect sensitivity and specificity were obtained then the curve would appear as a right angle. AUC was estimated using an asymptotically exact method (DeLong’s Method), which evaluates the uncertainty of the area under the curve.

Generalised variance inflation factors (GVIF) and adjusted GVIF were examined to determine the presence of multicollinearity. (Adjusted GVIF accounts for the dimensionality of the confidence ellipsoid and equals (GVIF)^(1/(2df)) where df denotes degree of freedom associated with the factor) [60].

Results are displayed in terms of odds ratios (OR) and 95 % confidence intervals (CI). ORs have a range from 0 to infinity. A value of 1.0 means there is no difference in odds. ORs greater than 1.0 indicate that the ratio of those with poor SRH versus good SRH in the selected group is greater than the baseline group. If the 95 % CI for OR contains 1 in the interval this indicates that at the 5 % significance level, there is no evidence to suggest that the ratio of those with poor SRH (versus good SRH) for the selected group are different from the baseline group.

All statistical analysis was performed using statistical software R (version 3.2.2) [61] and software packages lme4 (Linear Mixed-Effects Models using 'Eigen' and S4) [62] package pRoc (Display and Analyze ROC Curves) [63] and package car (Companion to Applied Regression) [60].

## Results

### Response rate

Of the 420 households invited to participate in the survey 343 were interviewed. This is a response rate of 81.6 %. The remainder were either un-contactable, or refused for reasons such as they did not have time to take part or had no interest in the research.

### Descriptive analyses

A demographic and socioeconomic description of the primary carers is reported (Table 1). The majority of primary carers were female, aged between 50 and 64 years of age, of Irish nationality, married, with mid-second level education, working full time, in receipt of a medical card, physically active and had experienced stress in the previous 12 months. Nearly half of primary carers indicated their health as being ‘good’ (*n* = 158/342; 46.2 %).

**Table 1:** Demographic and socioeconomic characteristics of the primary carer (*N* = 343)

Indicator	Number	%
Gender (*N* = 343/343; 100 %)		
Female	237	69.1 %
Male	106	30.9 %
Age (*N* = 339/343; 98.8 %)		
20–34	65	19.2 %
35–49	93	27.4 %
50–64	113	33.3 %
65+	68	20.1 %
Nationality (*N* = 325/343; 94.8 %)		
Irish	298	91.7 %
Other	27	8.3 %
Marital status (*N* = 342/343; 99.7 %)		
Married	179	52.3 %
Separated, divorced, widowed	72	21.1 %
Single	67	19.6 %
Cohabitating	24	7.0 %
Highest level of education attained (*N* = 337/343; 98.3 %)		
Primary education or less	90	26.7 %
Junior or intermediate certificate, technical or vocational training	75	22.3 %
Leaving certificate, A-level	55	16.3 %
Non-degree qualification	69	20.5 %
Degree, professional qualification or both	41	12.2 %
Postgraduate qualification	7	2.1 %
Current employment status (*N* = 343/343; 100 %)		
Working full time	94	27.4 %
Retired	71	20.7 %
Working in the home	54	15.7 %
Unemployed	51	14.9 %
Working part time	47	13.7 %
Ill/unable to work	19	5.5 %
In education	6	1.7 %
Government Employment Programme	1	0.3 %
Level of health cover (*N* = 341/343; 99.4 %)		
Medical card^a^	187	54.8 %
Neither medical card nor private health insurance	99	29.0 %
Private health insurance	47	13.8 %
Doctor visit card	8	2.3 %
Physical activity IPAQ score (*N* = 324/343; 94.5 %)		
Active	202	62.3 %
Inactive	122	37.7 %
Stress experienced in the last 12 months? (*N* = 341/343; 99.4 %)		
No	111	32.6 %
Yes	230	67.4 %
Self-rating of health (*N* = 342/343; 99.7 %)		
Very good	84	24.6 %
Good	158	46.2 %
Fair	76	22.2 %
Bad	19	5.6 %
Very bad	5	1.5 %

^a^State provided general medical (primary care) services (GMS) to those households on low income

A description of household occupancy status, number of people living within each household, whether there is a person with a chronic illness, a disability, and a smoker within the household is also reported, alongside healthcare utilisation in the previous 12 months (Table 2).

**Table 2:** Characteristics of the households (*N* = 343)

Indicator	Number	(%)
Household occupancy status (*N* = 336/343; 98.0 %)		
Outright owner/ Mortgage	203	60.4
Renting privately	29	8.6
Renting from or rent paid by health board/county council	104	31.0
Number of people living in the household (*N* = 341/343; 99.4 %)		
1 to 2 people	140	41.1
3 to 4 people	142	41.6
5 to 11 people	59	17.3
Median	3.0	
Does a person with a chronic illness live in the household? (*N* = 335/343; 97.7 %)		
No	161	48.1
Yes	174	51.9
Does a person with a disability live in the household? (*N* = 330/343; 96.2 %)		
No	272	82.4
Yes	58	17.6
Does a smoker live in the household? (*N* = 340/343; 99.1 %)		
No	189	55.6
Yes	151	44.4
Has a member of the household used the local hospital in the previous 12 months? (*N* = 342/343; 99.7 %)		
No	149	43.6
Yes	193	56.4
Did anyone in the household use general practice services in the previous 12 months? (*N* = 341/343; 99.4 %)		
No	18	5.3
Yes	323	94.7

### Regression analyses

Seventy-three household responses contained at least one missing observation. Thus, the complete data set was composed of 270 household responses. Table 3 shows the counts of poor SRH and good SRH for each variable, which corresponds to the data pertaining to the logistic regression analyses. Table 4 shows the characteristics of missing data for primary carers with poor SRH and good SRH.

**Table 3:** Characteristics of primary carers with poor SRH and good SRH

Note: 1 missing value for SRH	Good SRH (*N* = 242)	%	Poor SRH (*N* = 100)	%
Gender				
Male	71	29.3 %	35	35.0 %
Female	171	70.7 %	65	65.0 %
Age				
20–34	51	21.1 %	14	14.0 %
35–49	73	30.2 %	20	20.0 %
50–64	74	30.6 %	39	39.0 %
65+	41	16.9 %	26	26.0 %
Missing	3	1.2 %	1	1.0 %
Marital				
Married	129	53.3 %	50	50.0 %
Single	51	21.1 %	16	16.0 %
Co-habiting	17	7.0 %	7	7.0 %
Separated/divorced/widowed	44	18.2 %	27	27.0 %
Missing	1	0.4 %	0	0.0 %
Education				
Third level	97	40.1 %	20	20.0 %
Primary	51	21.1 %	38	38.0 %
Junior certificate^a^	56	23.1 %	19	19.0 %
Leaving certificate^b^	36	14.9 %	19	19.0 %
Missing	2	0.8 %	4	4.0 %
Employment				
Employed	124	51.2 %	18	18.0 %
Unemployed	66	27.3 %	39	39.0 %
Retired, ill or unable to work	52	21.5 %	43	43.0 %
Missing	0	0.0 %	0	0.0 %
Health Cover				
Private health insurance	41	16.9 %	6	6.0 %
Medical card^c^	115	47.5 %	79	79.0 %
None	84	34.7 %	15	15.0 %
Missing	2	0.8 %	0	0.0 %
Occupancy				
Owner/mortgage	155	64.0 %	47	47.0 %
Renting privately	18	7.4 %	11	11.0 %
Renting – council^d^	65	26.9 %	39	39.0 %
Missing	4	1.7 %	3	3.0 %
Number people in household				
1	25	10.3 %	22	22.0 %
2	61	25.2 %	31	31.0 %
3–5	128	52.9 %	39	39.0 %
6+	27	11.2 %	7	7.0 %
Missing	1	0.4 %	1	1.0 %
Stress				
No	90	37.2 %	21	21.0 %
Yes	151	62.4 %	79	79.0 %
Missing	1	0.4 %	0	0.0 %
Chronic Illness				
No	138	57.0 %	23	23.0 %
Yes	100	41.3 %	73	73.0 %
Missing	4	1.7 %	4	4.0 %
Disability				
No	205	84.7 %	66	66.0 %
Yes	27	11.2 %	31	31.0 %
Missing	10	4.1 %	3	3.0 %
Smoker				
No	138	57.0 %	50	50.0 %
Yes	102	42.1 %	49	49.0 %
Missing	2	0.8 %	1	1.0 %
IPAQ				
Active	146	60.3 %	56	56.0 %
Inactive	80	33.1 %	41	41.0 %
Missing	16	6.6 %	3	3.0 %
Local hospital use				
No	121	50.0 %	28	28.0 %
Yes	120	49.6 %	72	72.0 %
Missing	1	0.4 %	0	0.0 %
GP use				
No	16	6.6 %	2	2.0 %
Yes	224	92.6 %	98	98.0 %
Missing	2	0.8 %	0	0.0 %
Social Cohesion				
No	122	50.4 %	60	60.0 %
Yes	117	48.3 %	37	37.0 %
Missing	3	1.2 %	3	3.0 %

^a^Junior or intermediate certificate, technical or vocational training

^b^Leaving certificate, A-Level

^c^State provided general medical (primary care) services (GMS) to those households on low income

^d^Renting from or rent paid by health board/county council

**Table 4:** Characteristics of missing observations for primary carers with poor SRH and good SRH (*n* = 73)

	Good SRH (*N* = 49)	(%)	Poor SRH (*N* = 23)	(%)
Gender				
Male	16	32.7 %	14	60.9 %
Female	33	67.3 %	9	39.1 %
Unknown SRH (1 female)				
Age				
20–34	12	24.5 %	3	13.0 %
35–49	14	28.6 %	7	30.5 %
50–64	15	30.6 %	7	30.5 %
65+	5	10.2 %	5	21.7 %
Unknown age	3	6.1 %	1	4.3 %
Unknown SRH (1 age 65+)				
Marital				
Married	27	55.1 %	13	56.5 %
Single	12	24.5 %	4	17.4 %
Co-habiting	3	6.1 %	1	4.3 %
Separated/divorced/widowed	6	12.2 %	5	21.7 %
Unknown marital status	1	2.0 %	0	0.0 %
Unknown SRH (1 divorce/separated/widowed)				
Education				
Third level	16	32.7 %	9	39.1 %
Primary	13	26.5 %	4	17.4 %
Junior cert^a^	13	26.5 %	4	17.4 %
Leaving cert^b^	5	10.2 %	2	8.7 %
Unknown education	2	4.1 %	4	17.4 %
Unknown SRH (1 primary)				
Employment				
Employed	20	40.8 %	6	26.1 %
Unemployed	17	34.7 %	9	39.1 %
Retired, ill or unable to work	12	24.5 %	8	34.8 %
Unknown SRH (1 retired/ill/ unable to work)				
Health Cover				
Private	7	14.3 %	3	13.0 %
Medical card^c^	25	51.0 %	17	74.0 %
None	15	30.6 %	3	13.0 %
Unknown health cover	2	4.1 %	0	0.0 %
Unknown SRH (1 unemployed)				
Occupancy				
Owner/mortgage	28	57.1 %	10	43.5 %
Renting privately	1	2.0 %	1	4.4 %
Renting (council)^d^	16	32.7 %	9	39.1 %
Unknown occupancy	4	8.2 %	3	13.0 %
Unknown SRH (1 owner/mortgage)				
Number people in household				
1	5	10.2 %	4	17.4 %
2	6	12.3 %	7	30.5 %
3-5	31	63.3 %	10	43.5 %
6+	6	12.2 %	1	4.3 %
Unknown number of people in household	1	2.0 %	1	4.3 %
Unknown SRH (1 one person in household)				
Stress				
No	15	30.6 %	3	13.0 %
Yes	33	67.4 %	20	87.0 %
Unknown stress	1	2.0 %	0	0.0 %
Unknown SRH (1 unknown stress)				
Chronic Illness				
No	24	49.0 %	8	34.8 %
Yes	21	42.9 %	11	47.8 %
Unknown chronic illness	4	8.1 %	4	17.4 %
Unknown SRH (1 ‘yes’ chronic illness)				
Disability				
No	34	69.4 %	15	65.2 %
Yes	5	10.2 %	5	21.7 %
Unknown disability	10	20.4 %	3	13.0 %
Unknown SRH (1 ‘no’ disability)				
Smoker				
No	31	63.3 %	11	47.8 %
Yes	16	32.6 %	11	47.8 %
Unknown smoker status	3	6.1 %	1	4.4 %
Unknown SRH (1 ‘no’ smoker)				
IPAQ				
Active	22	44.9 %	13	56.6 %
Inactive	11	22.4 %	7	30.4 %
Unknown IPAQ	16	32.7 %	3	13.0 %
Unknown SRH (1 active IPAQ)				
Local hospital use				
No	24	49.0 %	6	26.1 %
Yes	24	49.0 %	17	73.9 %
Unknown local hospital use	1	2.0 %	0	0.0 %
Unknown SRH (1 ‘yes’ local hospital use)				
GP use				
No	4	8.2 %	0	0.0 %
Yes	43	87.7 %	23	100.0 %
Unknown GP use	2	4.1 %	0	0.0 %
Unknown SRH (1 ‘yes’ GP use)				
Social Cohesion				
No	23	46.9 %	8	34.8 %
Yes	23	46.9 %	12	52.2 %
Unknown social cohesion	3	6.1 %	3	13.0 %
Unknown SRH (1 ‘no’ SRH)				

^a^Junior or intermediate certificate, technical or vocational training

^b^Leaving certificate, A-Level

^c^State provided general medical (primary care) services (GMS) to those households on low income

^d^Renting from or rent paid by health board/county council

Results from the logistic regression model including random effect indicated that the unexplained variation within each cluster had a very low estimated standard deviation (<0.0001). In the principle of parsimony the logistic regression model without a random effect is adequate. This model gives results (effect estimates and standard error values) that are nearly the same as the results of the logistic regression model which includes a random effect. (The deviance between the logistic regression model and the logistic model including a random effect increases by 0.02. This difference in deviance is approximately chi-squared with one degree of freedom (*p*-value = 0.45, note: this *p*-value for comparing both models is half the *p*-value obtained from treating deviance as chi-squared with 1° of freedom) [59]. AIC for the random effects model is 269.27 and is 267.23 for the simplified model with variance = 0. Similarly BIC for the random effects model is 373.63 and is 368.03 for the less complex model.

The null deviance, which denotes the null model with just the constant term, was 322.8 and residual deviance was 220.22. This gives a difference of 102.58, with degrees of freedom = 19. Thus, the fitted model is more informative than the null model.

The issue of multicollinearity was not present as all adjusted GVIF values had a value less than 2. Table 5 shows results of the logistic regression model (with no random effects). Crude and adjusted odds ratios for primary carers with poor SRH compared with those with good SRH are displayed. ORs were based on the complete data set (where missing values were omitted from the analysis).

**Table 5:** Factors associated with poor self-rated health

	Crude OR	95 % CI	Adjusted OR^a^	95 % CI
Age group (years)				
20–34	1.28	(0.51, 3.15)	2.81	(0.87, 9.46)
35–49	Base		Base	
50–64	2.46	(1.20, 5.29)	1.67	(0.55, 5.32)
65+	2.65	(1.20, 6.05)	1.41	(0.33, 6.05)
Gender				
Male	Base		Base	
Female	0.78	(0.45, 1.39)	0.67	(0.32, 1.41)
Marital Status				
Married	Base			
Single	0.85	(0.39, 1.76)		
Co-habiting	1.18	(0.39, 3.18)		
Separated, divorced or widowed	1.60	(0.83, 3.03)		
Education				
Third level	Base		Base	
Primary education	6.59	(3.10, 14.98)	3.96	(1.44, 11.63)
Junior certificate^b^	2.57	(1.09, 6.21)	3.25	(1.09, 10.09)
Leaving certificate^c^	4.04	(1.73, 9.83)	3.89	(1.38, 11.57)
Employment				
Employed	Base		Base	
Unemployed	5.31	(2.56, 11.61)	3.91	(1.56, 10.25)
Retired, ill or unable to work	7.58	(3.67, 16.62)	4.06	(1.49, 11.61)
Health Cover				
Private health insurance	Base			
Medical card^d^	7.81	(1.90, 33.42)		
No health cover	1.97	(0.58, 9.06)		
Occupancy				
Outright owner/ Mortgage	Base		Base	
Renting privately	2.02	(0.83, 4.72)	5.09	(1.36,19.83)
Renting - Council^e^	2.10	(1.17, 3.77)	3.09	(1.31, 7.62)
Number of people in household				
1	3.01	(1.41, 6.47)	2.77	(0.91, 8.72)
2	1.46	(0.77, 2.75)	0.95	(0.40, 2.22)
3–5	Base		Base	
6+	0.96	(0.33, 2.47)	0.28	(0.07, 1.00)
Stress				
No	Base			
Yes	2.08	(1.16, 3.89)	1.35	(0.63, 2.92)
Chronic illness				
No	Base		Base	
Yes	5.96	(3.24, 11.57)	4.78	(2.09, 11.64)
Disability				
No	Base			
Yes	3.96	(2.08, 7.64)	2.07	(0.89, 4.84)
Smoker				
No	Base			
Yes	1.21	(0.71,2.06)		
IPAQ				
Active	Base			
Inactive	1.42	(0.83, 2.43)		
Local hospital use				
No	Base		Base	
Yes	2.52	(1.45, 4.53)	2.01	(1.00, 4.14)
Household GP use				
No	Base			
Yes	2.49	(0.66, 16.22)		
Social cohesion score				
5 or less	Base			
6-10	0.51	(0.29, 0.87)	0.52	(0.25, 1.05)

^a^Logistic regression – adjusting for other factors included in the model

^b^Junior or intermediate certificate, technical or vocational training

^c^Leaving cert, A-Level

^d^State provided general medical (primary care) services (GMS) to those households on low income

^e^Renting from or rent paid by health board/county council

**Fig. 2:**
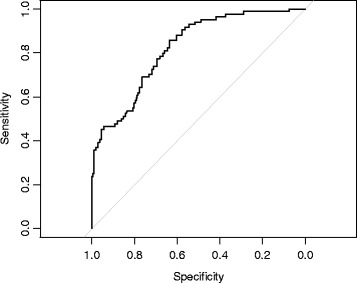
Receiver operating curve to display the accuracy of logistics regression model

Model evaluations indicate that this model is accurate in terms of sensitivity and specificity (see Fig. 2 for corresponding ROC curve). AUC was estimated to be 0.85 (95 % CI: 0.79–0.89).

Statistically significant factors include level of education, employment, occupancy, number of people living in household, chronic illness and local hospital use. Adjusting for the effects of other factors, the odds of primary carers who had primary education (OR 3.96, 95 % CI (1.44, 11.63)), junior certificate (or intermediate certificate, technical or vocational training) (OR 3.25, 95 % CI (1.09, 10.09)) or leaving certificate (A-levels or technical qualification) (OR 3.89 95 % CI (1.38, 11.57)) were greater to have poor self-rated health in comparison to primary carer’s with third level education. The odds of primary carers who were renting privately (OR 5.09, 95 % CI (1.36, 19.83)) or renting from, or having rent paid in by the health board/county council (OR 3.09, 95 % CI (1.31, 7.62)) were increased for poor SRH in comparison to the odds to self-rated health than primary carers who owned their own property outright or who had a mortgage. The odds of primary carers who were unemployed (OR 3.91, 95 % CI (1.56, 10.25)) or retired, ill or unable to work (OR 4.06, 95 % CI (1.49, 11.61)) were greater for having poor SRH than the odds of primary carers who had employment. If any resident of the household had a chronic illness then the odds of a primary carer having poor self-rated health was greater than the odds for a primary carer in a household where no resident had a chronic illness (OR 4.78, 95 % CI (2.09, 11.64). If any resident of the household used the local hospital, the primary carer had greater odds of having poor self-rated health than the odds for a primary carer in a household where no resident used the local hospital (OR 2.01, 95 % CI (1.00, 4.14)).

## Discussion

Self-rating of health (SRH) is affected by both health and non-health related factors. Nearly half of the primary carers in the current study reported their heath as ‘good’. Of those that reported their health as being ‘poor’ factors, such as, educational attainment, employment status, whether a person owns their own home, whether there was someone living in the household with a chronic illness and whether any of the household residents had used the local hospital, had a role to play in the primary carer reporting poorer SRH.

While there is a growing understanding of the role of SRH, a paradigm model has yet to be widely accepted with recent studies concluding that further work is required in determining whether there are important predictors of SRH yet to be highlighted [1, 50]. The relationship between the biological and physiological mechanisms through which SRH results in chronic disease in the presence of physical, psychosocial and environment stressors has been explored [64]. A further consideration is the need to control for differences in reporting behaviour. A growing literature shows that respondents tend to evaluate their health differently according to a number of non-health characteristics [33, 49, 65], including age, gender and education and failure to account for these factors may bias the estimated associations between health dimensions and SRH [1].

Self-rating of health is commonly used in large population level research due to its ease of use and its power in measuring health. The same questions on self-rating of health were put to primary carers in the 2014 HANA survey and to all people living within each household in the 2011 National Census within the same geographical area. Respondents in the HANA survey gave a lower rating of their overall health status (70.8 % reporting ‘very good’ or ‘good’ health status) than respondents from the same electoral divisions during the census (89.9 %). The disparity between the two time points in how participants rank their health may be due to the greater focus on health in the survey in 2014 as opposed to the 2011 national census which asks a broad range of questions, such as whether the house had piped water and sewage facilities and access to the internet. Alternatively, this difference could be due to the national census being self-completed and the HANA survey being researcher administered.

We found no evidence to support the role of age, gender and marital status in SRH, which would differ from previously published research [11, 66, 67].

Neither attendance at a general practitioner nor social cohesion was related to SRH. This is contrary to other studies conducted in Ireland and internationally. An Irish study based on SLAN in 2002 reported that high levels of social trust independently reduced the risk of reporting poor mental health [68]. An evaluation of social cohesion and self rating of health using data from 29 high income countries from the 2000 World Value Survey also found an association between social capital and good health [69]. The difference in findings in the current research may be as a result of all participants being resident within the same area and being more likely to have a similar level of social cohesion.

The odds of primary carers reporting that there was someone in their home with a chronic illness were nearly 5 times greater for rating their health as poor than the odds of a primary carer who did not report that there was a person with a chronic illness in the household. This is consistent with other research which has demonstrated a link between SRH and people living with chronic diseases such as CVD [32], arthritis, diabetes [31], lung disease and stroke [26]. The way that the question was asked in the HANA survey means that we are unable to determine whether it was the primary carers themselves with a chronic illness. In future research it would be of interest to delineate whether a lower self-rating of health occurs from the simple presence of having a person with a chronic illness in the house rather than the respondent themselves having the chronic condition and that affecting their SRH.

Unemployed, retired or ill primary carers were more likely to indicate lower self-rating of health when compared to employed people. This relationship between self-rated health and employment status is stronger than that which has previously been reported in the literature. A systematic review of the factors in the trajectory of self-rating of health identified a moderate relationship between self-rating of health and employment status [70] while a study of patients with multiple sclerosis reported those with good self-rated health were 2.46 times more likely to be in employment [71]. The association between SRH and employment may be greater in this study due to the effects of a recent economic recession resulting in high levels of unemployment, particularly in deprived areas.

The odds of a primary carer who reported renting privately were five times greater, and the odds of a primary carer who reported renting through social welfare support, were three times greater to rate their health as poorer compared to the odds of those who indicated that they are the out-right owner or mortgage holder of their home. This adds to current literature which identifies the link between poorer self-rating of health and occupancy status, particularly for those who are renters [72] although the strength of this link does not appear to have been previously identified.

In the current study, SRH was not associated with stress, gender or being a smoker. The number of stressful life events experienced is associated with poorer SRH [73]. A study of black American adults reported a significant association between SRH and stress [74]. In the current study, stress was significantly associated with SRH using crude 0R but not significant using adjusted ORs. A recent systematic review examining factors of change and cumulative factors in SRH found that gender is only moderately associated with growing influences of SRH factors over time [70]. Furthermore, the authors found that being a man was an advantageous factor, but the strength and direction of the association was inconsistent; with the influence of gender being weak and consistent in some studies and strong with minor inconsistency in other studies. Smoking more than 100 cigarettes in an entire life span is associated with lower SRH in two studies with adolescents and Asian American adults [75, 76]. Contrary to this, research conducted in Spain and Catalonia did not find an association between smoking status and SRH [77]. Also it is important to note that in the current study caution must be exercised when interpreting results as 73/343 houses had a least one missing value. This accounts for approximately 21 % missing data.

This study had a very good response rate of 82 % and replicated a study completed in the same area in 2001 by employing a random cluster sampling method to select households. Standardised questions were included where possible. We conducted a media awareness campaign and rigorously trained experienced interviewers to strengthen the methodology. The steering committee for the research included partnerships with a number of community groups working in the area.

Consideration must be given to the fact that the sample represents 13 electoral divisions in one deprived suburb of Dublin and is not nationally representative. However, the inclusion of questions such as social capital and SRH complimentary to European studies [9] and the Irish national census [15] allow the data to be compared in national and European contexts. Previous studies have highlighted a need for such data collected at the community level, rather than consistently at the aggregated population level [78] so that data can be mobilised and utilised as part of health improvement strategies at local level. Data was collected in the current study through face-to-face interviewer administrated questionnaires. A weakness of previous research in the Republic of Ireland is that it has relied on self-completed postal questionnaires and had a lower response rate; and thus, it is possible that non-participants in the survey may have had differing patterns of health that would have impacted on the results [50].

This study employed a logistic regression model to investigate the relative importance of the effects of different factors on respondent’s SRH. However, this approach requires that all relevant factors are included in the set of variables entered into the multiple regression and that the different factors are all measured on a comparable scale. Failure to account for non-health characteristics may bias the estimated associations between health factors and SRH and this will need to be considered.

A recent systematic review has highlighted the need for future work to focus on the effects of unemployment on SRH within subsections of a population, rather than in the population as a whole as has been done in the past [79, 80]. It would be particularly interesting to track potential changes in SRH as people return to employment following a deep recession in Ireland.

## Conclusion

Health planners and policy makers are increasingly asking for a feasible method to identify vulnerable persons with the greatest health needs. SRH is an easy to administer question at population level that can assist in identifying persons at risk of poor health. Limitations aside, this study demonstrates that educational attainment, employment status, household occupancy, numbers of people living within the household, chronic illness and local hospital use are significant in predicting whether a person rates their health as ‘poor’ or ‘good’. Future health and social policies should be cognisant of the impact of both health and non-health related factors on perceived health status.

## Abbreviations

AUC, area under the curve; CI, confidence interval; CVD, cardiovascular disease; DI, deprivation Index; ED, electoral division; HANA, health assets and needs assessment; IPAQ, international physical activity questionnaire; OECD, organisation for economic cooperation and development; OR, odds ratio; ROC Curve, receiver operating characteristic curve; SAHRU, Small Area Health Research Unit; SLAN, survey of lifestyle, attitudes and nutritional survey; SRH, self-rated health

## Additional file

Additional file 1: Copy of survey instrument. (DOCX 250 kb)
