# Supplementary material for: Determinants of self-rated health in an Irish deprived suburban population – a cross sectional face-to-face household survey
Source: BMC Public Health. 2016 Aug 11;16:767. doi: 10.1186/s12889-016-3442-x (PMC4982417; doi:10.1186/s12889-016-3442-x)
Supplement: Additional file 1: — Copy of survey instrument. (DOCX 250 kb) [file 12889_2016_3442_MOESM1_ESM.docx]

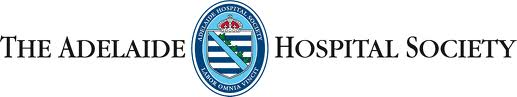

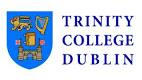

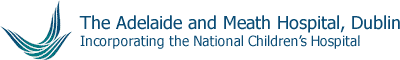


**Health Assets and Needs Assessment Tallaght**

**(HANA in Tallaght)**

An assessment of health assets, needs and health service satisfaction reported by the population of Tallaght 2014

**4 Person Household Questionnaire**

**Household ID: ___________________________**

**Interviewer: _____________________________**

**Date: _________________________**

**Instructions**

- The primary carer is the person in the household who manages the welfare and health of the family/household. In a house of renters this is the person who pays the bills or whose name is on the rent agreement.
- Unless otherwise specified all questions are to be answered by and in relation to the primary carer.
- Unless otherwise specified tick one answer only for each question.

**Part 1: Demographics**

**Section One: Household Demographic details**

**1.1** Complete the following about the primary carer.

***(Tick one only in each line)***

| **Primary Carer Name: ____________________________** | |
| --- | --- |
| Gender | ▢ Male ▢ Female |
| What age are you? |  |
| Nationality |  |
| Marital status | ▢ Single ▢ Cohabiting ▢ Married ▢ Separated, divorced, widowed |
| Highest level of education attained | ▢ Primary education or less  ▢ Junior or intermediate certificate, technical/vocational training  ▢ Leaving certificate, A level and technical training  ▢ Non degree qualification (diploma, certificate)  ▢ Degree, professional qualification or both  ▢ Postgraduate qualification |
| What is your current employment status?  ***(Tick all which apply)*** | ▢ Working full time ▢ Working part time ▢ In education ▢ Job Bridge  ▢ Working in the home ▢ Ill/unable to work ▢ Unemployed ▢ Retired  ▢ Unpaid voluntary work experience |
| What is your job?  ***(For those working full or part time only)*** |  |
| What health cover do **you** currently have?  ***(Tick all which apply)*** | ▢ Medical card/GMS card ▢ Doctor visit card  ▢ Private medical insurance ▢ Neither medical card nor private insurance |
| How many years have you lived in this house? |  |
| House Occupancy Status | ▢ Outright owner ▢ Tenant purchasing plan ▢ Renting privately  ▢ Mortgage ▢Renting from or rent paid by health board/county council |
| Do you own a car? | ▢ Yes ▢ No |

**1.2** How many people live here (**including primary carer**)? _____

**1.3** Complete the following for each person living in the household (**excluding the primary carer**).

***(Tick one only in each line)***

| **Person 1 Name: ______________________________________** | |
| --- | --- |
| Gender | ▢ Male ▢ Female |
| What age are they? |  |
| Relationship with primary carer | ▢ Spouse/Partner ▢ Child ▢ Grandchild ▢ Parent  ▢ Not related ▢ Other ____________ |
| Current employment status  ***(Tick all which apply)*** | ▢ Working full time ▢ Working part time ▢ In education ▢ Job Bridge  ▢ Working in the home ▢ Ill/unable to work ▢ Unemployed ▢ Retired  ▢ Unpaid voluntary work experience |

***(Tick one only in each line)***

| **Person 2 Name: ______________________________________** | |
| --- | --- |
| Gender | ▢ Male ▢ Female |
| What age are they? |  |
| Relationship with primary carer | ▢ Spouse/Partner ▢ Child ▢ Grandchild ▢ Parent  ▢ Not related ▢ Other ____________ |
| Current employment status  ***(Tick all which apply)*** | ▢ Working full time ▢ Working part time ▢ In education ▢ Job Bridge  ▢ Working in the home ▢ Ill/unable to work ▢ Unemployed ▢ Retired  ▢ Unpaid voluntary work experience |

***(Tick one only in each line)***

| **Person 3 Name: ______________________________________** | |
| --- | --- |
| Gender | ▢ Male ▢ Female |
| What age are they? |  |
| Relationship with primary carer | ▢ Spouse/Partner ▢ Child ▢ Grandchild ▢ Parent  ▢ Not related ▢ Other ____________ |
| Current employment status  ***(Tick all which apply)*** | ▢ Working full time ▢ Working part time ▢ In education ▢ Job Bridge  ▢ Working in the home ▢ Ill/unable to work ▢ Unemployed ▢ Retired  ▢ Unpaid voluntary work experience |

**Part 2: Health Needs**

**Section 2: Health status**

**2.1** How would you rate **your** health?

▢Very bad ▢ Bad ▢ Fair ▢ Good ▢ Very good

**Stress**

**2.2** Have **you** experienced stress within the last 12 months? ▢ Yes ▢ No ***If no skip to question 2.4***

**2.3** Complete the following table

***(Tick one only in each unless otherwise specified)***

| Thinking about the stress you have experienced, what was the reason for this stress? |  |
| --- | --- |
| How would you rate the **seriousness** of this stress? | ▢ 1 ▢ 2 ▢3 ▢ 4 ▢ 5  ***Not serious Very serious*** |
| Which of the following have you experienced as a result of stress in the last 12 months?  ***(Tick all which apply)*** | ▢ Anxious ▢ Depressed ▢ Eating too much/too little  ▢ Annoyed ▢ Illness ▢ Smoke more  ▢ Aggressive ▢ Sleeplessness ▢ Take more alcohol/drugs ▢ None ▢ Other ________________________________ |
| Which of the following actions have you taken as a result of stress that you have experienced in the last 12 months?  ***(Tick all which apply)*** | ▢ Visited counsellor/psychiatrist/psychologist  ▢ Taken prescription medication ▢Visited church  ▢ Visited GP ▢ Talked to friends/relatives  ▢ None ▢ Other _______________ |

**2.4** How many teenagers in your household are currently aged between 13 and 19 years of age? _____

***If none skip to question 2.6***

**2.5** Many parents have difficulties coping with children during their teenage years, we would like to ask your current experience of the teenage children in this household. For each teenager between the age of 13 and 19 years of age complete the following table.

***(Tick one only in each unless otherwise specified)***

| **Name (Teenager 1): __________________________________** | |
| --- | --- |
| Do you worry about X (teenagers name) when s/he socialises? | ▢ Yes ▢ No  ***If no skip to line 3 of box*** |
| **Why** do you worry about X (teenagers name) when s/he socialises? |  |
| Are you happy with X (teenagers name) friends? | ▢ Yes ▢ No ▢ Don't know their friends |
| Have you found X (teenagers name) attitude or behaviour problematic in the last 12 months? | ▢ Yes ▢ No ***If no skip to question 2.6*** |
| What action or behaviour (by your teenager) have you found most problematic in the last 12 months? | ▢ Violent/aggressive episodes ▢Unmanageable  ▢ Takes/sells drugs or alcohol ▢ Dieting  ▢ Refuses to go to school/study ▢ Mood swings  ▢ None ▢Other ___________ |
| Where have **you** gone for help **for yourself** about your teenagers behaviour in the last 12 months?  ***(Tick all which apply****)* | ▢ Family ▢ Teacher ▢ GP ▢ Social/youth worker  ▢ Friend ▢ Church ▢ Counsellor ▢ Garda  ▢ None ▢Other_______________ |

***(Tick one only in each unless otherwise specified)***

| **Name (Teenager 2): __________________________________** | |
| --- | --- |
| Do you worry about X (teenagers name) when s/he socialises? | ▢ Yes ▢ No  ***If no skip to line 3 of box*** |
| **Why** do you worry about X (teenagers name) when s/he socialises? |  |
| Are you happy with X (teenagers name) friends? | ▢ Yes ▢ No ▢ Don't know their friends |
| Have you found X (teenagers name) attitude or behaviour problematic in the last 12 months? | ▢ Yes ▢ No ***If no skip to question 2.6*** |
| What action or behaviour (by your teenager) have you found most problematic in the last 12 months? | ▢ Violent/aggressive episodes ▢Unmanageable  ▢ Takes/sells drugs or alcohol ▢ Dieting  ▢ Refuses to go to school/study ▢ Mood swings  ▢ None ▢Other ___________ |
| Where have **you** gone for help **for yourself** about your teenagers behaviour in the last 12 months?  ***(Tick all which apply****)* | ▢ Family ▢ Teacher ▢ GP ▢ Social/youth worker  ▢ Friend ▢ Church ▢ Counsellor ▢ Garda  ▢ None ▢Other_______________ |

**Disabilities**

**2.6** How many people in this household, if any, are in receipt of a disability allowance? _____

**Chronic Illness**

***Use laminate provided***

**2.7** How many people in this household have a ***chronic illness*** *? _____ ***If none skip to question 2.9***

*A chronic illness is an illness that has been present for some time or recurs frequently requiring medical treatment, see laminate for examples.

**2.8** Complete the following table for each person in the household who has a ***chronic illness***.

***(Tick one only in each unless otherwise specified)***

| **Name (Person 1 with a chronic illness):____________________** | |
| --- | --- |
| What chronic illness(es) does this person have? |  |
| Degree of care required | ▢ No assistance  ▢ Medication only  ▢ Housekeeping including medication  ▢ Housekeeping, medication and help to sit out in a chair  ▢ Total nursing care as confined to bed |
| Does X (name of person) have organised home-help? | ▢ Yes ▢ No ▢ NA |
| In the last three months was X (name of person) visited by a public health nurse in relation to their chronic illness? | ▢ Yes ▢ No ▢ NA |
| In the last three months did X (name of person) visit the GP in relation to their chronic illness? | ▢ Yes ▢ No ▢ NA  ***If no skip to last line of table*** |
| What was the reason for this/these GP visit (s)? | ▢ Repeat prescription ▢ Medical check up  ▢ Sudden illness ▢ Advice  ▢Other ______________ |
| In the last three months X (name of person) attend Tallaght hospital in relation to their chronic illness? | ▢ Yes ▢ No |

***(Tick one only in each unless otherwise specified)***

| **Name (Person 2 with a chronic illness):____________________** | |
| --- | --- |
| What chronic illness(es) does this person have? |  |
| Degree of care required | ▢ No assistance  ▢ Medication only  ▢ Housekeeping including medication  ▢ Housekeeping, medication and help to sit out in a chair  ▢ Total nursing care as confined to bed |
| Does X (name of person) have organised home-help? | ▢ Yes ▢ No ▢ NA |
| In the last three months was X (name of person) visited by a public health nurse in relation to their chronic illness? | ▢ Yes ▢ No ▢ NA |
| In the last three months did X (name of person) visit the GP in relation to their chronic illness? | ▢ Yes ▢ No ▢ NA  ***If no skip to last line of table*** |
| What was the reason for this/these GP visit (s)? | ▢ Repeat prescription ▢ Medical check up  ▢ Sudden illness ▢ Advice  ▢Other ______________ |
| In the last three months X (name of person) attend Tallaght hospital in relation to their chronic illness? | ▢ Yes ▢ No |

**Substance Use**

**2.9** How many people in your household smoke? _____

**2.10** How many people **under the age of 18** smoke in your household? _____

**2.11** Do you think that **anyone** in your household has or has had a problem with alcohol or drugs dependency **ever**?

***(Tick all which apply****)* ▢ Yes ▢ No ▢ Don't know ▢ Prefer to skip section

***If no, don't know or prefer to skip section skip to question 2.13***

**2.12** For each individual who has or has had a problem with alcohol or drugs complete the following table:

***(Tick one only in each unless otherwise specified)***

| **Name (Person 1 with substance problem): __________________________________** | |
| --- | --- |
| Scale of the problem | ▢ 1 ▢ 2 ▢3 ▢ 4 ▢ 5  ***Not serious Very serious*** |
| Main drug used | ▢ Alcohol ▢ Pain medication ▢ Heroin  ▢ Cannabis ▢ Illegal methadone ▢ Headshop products  ▢ Sedatives ▢ Stimulants ▢Other ___________ |
| As a result of X (name of person) substance use which of the following have **they** done?  ***(Tick all which apply)*** | ▢Talked to family/friends ▢Taken sedatives  ▢Visited the GP ▢Attended methadone maintenance  ▢Attended a support group ▢Attended methadone detox  ▢Attended counselling ▢Used needle exchange programme  ▢ None ▢Other ______________________ |

***(Tick one only in each unless otherwise specified)***

| **Name (Person 2 with substance problem): __________________________________** | |
| --- | --- |
| Scale of the problem | ▢ 1 ▢ 2 ▢3 ▢ 4 ▢ 5  ***Not serious Very serious*** |
| Main drug used | ▢ Alcohol ▢ Pain medication ▢ Heroin  ▢ Cannabis ▢ Illegal methadone ▢ Headshop products  ▢ Sedatives ▢ Stimulants ▢Other ___________ |
| As a result of X (name of person) substance use which of the following have **they** done?  ***(Tick all which apply)*** | ▢Talked to family/friends ▢Taken sedatives  ▢Visited the GP ▢Attended methadone maintenance  ▢Attended a support group ▢Attended methadone detox  ▢Attended counselling ▢Used needle exchange programme  ▢ None ▢Other ______________________ |

**Physical Activity**

**2.13** Consider a 7 day period (1 week). How many times on average do **you** do the following kinds of exercise for more than 20 minutes **during your free time** (not work related)?

**Strenuous exercise** (heart beats rapidly) _____ times

e.g. running, jogging, hurling, camogie, football, squash,basketball, judo,

vigorous swimming, vigorous long distance cycling, advanced aerobics

**Moderate exercise** (not exhausting) _____ times

e.g. fast walking, tennis, badminton, easy swimming, easy cycling,

intermediate aerobics, heavy gardening

**Mild exercise** (minimal effort) _____ times

e.g. yoga, golf, easy walking, bowling, beginners aerobics, light gardening.

**2.14** How many days, if any, in an average week do you walk for 30 minutes or more? _____ days

**Section 3: Tallaght Hospital**

**3.1** How many people in your household (including you) have had tests or treatment in Tallaght Hospital (**excluding A&E**) within the last 12 months? ___ ***If none skip to question 3.5***

**3.2** Complete the following table for each person who has had tests or received treatment in Tallaght Hospital (**excluding A&E**) within the last 12 months.

***(If more than one visit please describe the more recent visit. Tick one only in each unless otherwise specified)***

| **Name (Person 1 who received test or treatment): ___________________** | |
| --- | --- |
| Reason for attending Tallaght Hospital |  |
| Source of referral | ▢ Themselves ▢ GP ▢ Hospital Doctor |
| How would you rate your satisfaction with Tallaght Hospital?  ***If rated 4-6 please skip to last line of box*** | ▢ 1 ▢ 2 ▢3 ▢ 4 ▢ 5 ▢ 6  ***Dissatisfied***  ***Satisfied*** |
| If you rated Tallaght Hospital 1-3 what were your main reasons for dissatisfaction?  ***(Tick all which apply)*** | ▢ Lack of friendliness/respect/compassion provided by staff  ▢ Poor quality of care ▢ Hospital environment  ▢ Long waiting times ▢ Hospital cleanliness  ▢ Speed of care too slow ▢ Hospital safety  ▢ Speed of care too quick ▢ Other ________________ |
| Would you recommend Tallaght Hospital to a friend/family member? | ▢ Yes ▢ No ▢Don't know |

***(If more than one visit please describe the more recent visit. Tick one only in each unless otherwise specified)***

| **Name (Person 2 who received test or treatment): ___________________** | |
| --- | --- |
| Reason for attending Tallaght Hospital |  |
| Source of referral | ▢ Themselves ▢ GP ▢ Hospital Doctor |
| How would you rate your satisfaction with Tallaght Hospital?  ***If rated 4-6 please skip to last line of box*** | ▢ 1 ▢ 2 ▢3 ▢ 4 ▢ 5 ▢ 6  ***Dissatisfied***  ***Satisfied*** |
| If you rated Tallaght Hospital 1-3 what were your main reasons for dissatisfaction?  ***(Tick all which apply)*** | ▢ Lack of friendliness/respect/compassion provided by staff  ▢ Poor quality of care ▢ Hospital environment  ▢ Long waiting times ▢ Hospital cleanliness  ▢ Speed of care too slow ▢ Hospital safety  ▢ Speed of care too quick ▢ Other ________________ |
| Would you recommend Tallaght Hospital to a friend/family member? | ▢ Yes ▢ No ▢Don't know |

***(If more than one visit please describe the more recent visit. Tick one only in each unless otherwise specified)***

| **Name (Person 3 who received test or treatment): ___________________** | |
| --- | --- |
| Reason for attending Tallaght Hospital |  |
| Source of referral | ▢ Themselves ▢ GP ▢ Hospital Doctor |
| How would you rate your satisfaction with Tallaght Hospital?  ***If rated 4-6 please skip to last line of box*** | ▢ 1 ▢ 2 ▢3 ▢ 4 ▢ 5 ▢ 6  ***Dissatisfied***  ***Satisfied*** |
| If you rated Tallaght Hospital 1-3 what were your main reasons for dissatisfaction?  ***(Tick all which apply)*** | ▢ Lack of friendliness/respect/compassion provided by staff  ▢ Poor quality of care ▢ Hospital environment  ▢ Long waiting times ▢ Hospital cleanliness  ▢ Speed of care too slow ▢ Hospital safety  ▢ Speed of care too quick ▢ Other ________________ |
| Would you recommend Tallaght Hospital to a friend/family member? | ▢ Yes ▢ No ▢Don't know |

**3.3** Do **you** think that Tallaght Hospital is beneficial to the surrounding community?

▢ Yes ▢ No ▢Don't know ***If no skip to question 3.5***

**3.4** If yes, why do you think that Tallaght Hospital is beneficial to the surrounding community?

________________________________________________________________________________________________________________________________________________

**Tallaght Hospital A&E**

**3.5** Have anyone in your household (including you) used Tallaght Hospital A&E over the past 12 months?

▢ Yes ▢ No ▢Don't know ***If no or don't know skip to question 3.7***

**3.6** Thinking of the **most recent visit** to Tallaght Hospital **A&E** by anyone in your household (including you) in the past 12 months complete the following.

***(Tick one only in each unless otherwise specified)***

| **Name (Person 1 used Tallaght Hospital A&E): ___________________** | |
| --- | --- |
| How were you/they referred? | ▢ GP referral after attending ▢ Themselves  ▢ GP referral over the phone ▢ Came in by ambulance  ▢ Other __________________________ |
| If self-referral, why did you/they not go to see another healthcare professional, such as your GP, beforehand? | ▢ GP was not available ▢ GP Too expensive  ▢ GP didn't have access to same tests e.g. x-ray  ▢ Other________________________ |
| How long were you/they sick before attending A&E? | ▢ <24 hours ▢ 1-2 days ▢ 3-7 days ▢ 1-2 weeks  ▢ 2-4 weeks ▢ 1-2 months ▢ >2 months |
| What was your/their reason for attendance? |  |
| Would you recommend the Tallaght Hospital A&E to a friend/family member? | ▢ Yes ▢ No ▢Don't know |
| How would you rate Tallaght Hospital A&E? | ▢1 ▢ 2 ▢3 ▢ 4 ▢ 5 ▢ 6  ***Dissatisfied Satisfied*** |
| If you rated Tallaght Hospital A&E 1-3 what were you main reasons for dissatisfaction? | ▢ Lack of friendliness/respect/compassion provided by staff  ▢ Poor quality of care ▢ Hospital safety  ▢ Long waiting times ▢ Hospital cleanliness  ▢ Speed of care too slow ▢ Hospital environment  ▢ Speed of care too quick ▢ Other _________________ |

**Tallaght Hospital Waiting Lists**

**3.7** How many people in your household (including you) are on a waiting list to receive treatment in **Tallaght Hospital**? ___

***If none skip to question 3.9***

**3.8** For each individual in your household who is waiting for healthcare complete the following table:

***(Tick one on each line unless otherwise specified)***

| **Name (Person 1 waiting for healthcare): __________________________** | |
| --- | --- |
| Number of months on waiting list | ▢ 3 or less ▢ 4-6 ▢7-12 ▢ 13 or more |
| Type of treatment X waiting for |  |
| How reasonable would you describe this waiting time? | ▢ 1 ▢ 2 ▢3 ▢ 4 ▢ 5  ***very unreasonable very reasonable*** |

***(Tick one on each line unless otherwise specified)***

| **Name (Person 2 waiting for healthcare): __________________________** | |
| --- | --- |
| Number of months on waiting list | ▢ 3 or less ▢ 4-6 ▢7-12 ▢ 13 or more |
| Type of treatment X waiting for |  |
| How reasonable would you describe this waiting time? | ▢ 1 ▢ 2 ▢3 ▢ 4 ▢ 5  ***very unreasonable very reasonable*** |

**Future Services in Tallaght Hospital**

**3.9** Please comment on how you think **Tallaght Hospital** could improve the service it provides?

________________________________________________________________________________________________________________________________________________________________________________________________________________________

**3.10** Would you like to be more involved in the decisions Tallaght Hospital makes in changing and improving its services?

▢ Yes ▢ Yes, but unsure what difference it would make ▢ No ▢Don't know

**Section 4: General Practice and Healthcare Services**

**General Practice Services**

**4.1** Is your GP within walking distance of your house? ▢ Yes ▢ No ▢Don't know

**4.2** Would you recommend your GP to a friend/family member? ▢ Yes ▢ No ▢Don't know

**4.3** How would you rate your satisfaction with your GP? ▢ 1 ▢ 2 ▢3 ▢ 4 ▢ 5 ▢ 6

***Dissatisfied***  ***Satisfied***

**4.4** When anyone in your household (including you) need 'out of hours' doctor services what do you do? ***(Tick all which apply)***

▢ TLC Doc ▢ Go to A&E ▢ N/A

▢House call ▢Depends on situation ▢ Other ________________

**4.5** Are you satisfied with current 'out of hours' doctor service options? ▢ Yes ▢ No ▢Don't know

**General Healthcare Services**

**4.6** Where would you prefer to receive the following tests? ***Tick one only in each line***

| Test | GP | Hospital |
| --- | --- | --- |
| Blood test |  |  |
| X-ray |  |  |
| Ultra sound |  |  |

**4.7** What healthcare services are needed in the **Tallaght community**?

________________________________________________________________________________________________________________________________________________

**Part 3: Health Assets**

**Section 5: Personal and Community Characteristics**

**5.1** What are the top three **good things** about living in Tallaght?

1. __________________________________________________________________

2. __________________________________________________________________ 3. __________________________________________________________________

**5.2** What are the top three **bad things** about living in Tallaght?

1. __________________________________________________________________

2. __________________________________________________________________ 3. __________________________________________________________________

**5.3** Generally speaking, would you say that most people can be trusted, or that you can't be too careful in dealing with people?

Please tell me on a scale of 1 to 10, where 1 means that you can't be too careful and 10 means that most people can be trusted.

(**Tick one only)**

| 1  You can't be too careful | 2 | 3 | 4 | 5 | 6 | 7 | 8 | 9 | 10  Most people can be trusted |
| --- | --- | --- | --- | --- | --- | --- | --- | --- | --- |
|  |  |  |  |  |  |  |  |  |  |

**Section 6: Healthcare Inventory**

| **Frequency Last 12 Months** |
| --- |
| **Daily:** 4 to 7 times per week **Weekly:** 1 to 3 times per week **Monthly:** 7 to 12 times a year  **Bimonthly:** 3 to 6 times a year **Once or twice:** 1 or 2 times a year |

***Use laminates provided***

**General Practice Services**

**6.1** Did anyone in your household (including you) use GP services in the last 12 months?

▢ Yes, GP services **in** Tallaght used ▢ Yes, GP services **outside** of Tallaght used

▢ No, GP services not used ▢ Don't know

***If yes, GP services outside of Tallaght used skip to question 6.3***

***If no GP services not used or don't know skip to question 6.4***

**6.2** Thinking of GP services in Tallaght used in the last 12 months complete the following:

***(Tick one on each line only)***

| How often did your household use GP services in Tallaght in the last 12 months? | ▢ Daily ▢ Weekly ▢ Monthly  ▢ Bimonthly ▢ Once or twice |
| --- | --- |
| Are GP services in Tallaght an asset to the community? | ▢ Yes ▢ No |
| **If no,** please indicate **why not**? |  |

***(Tick one on each line only)***

| How often did your household use GP services in Tallaght in the last 12 months? | ▢ Daily ▢ Weekly ▢ Monthly  ▢ Bimonthly ▢ Once or twice |
| --- | --- |
| Are GP services in Tallaght an asset to the community? | ▢ Yes ▢ No |
| **If no,** please indicate **why not**? |  |

**6.3** Why did your household use GP services **outside** of Tallaght? __________________________________

________________________________________________________________________

| **Frequency Last 12 Months** |
| --- |
| **Daily:** 4 to 7 times per week **Weekly:** 1 to 3 times per week **Monthly:** 7 to 12 times a year  **Bimonthly:** 3 to 6 times a year **Once or twice:** 1 or 2 times a year |

**Pharmacy**

**6.4** Did anyone in your household (including you) use a pharmacy in the last 12 months?

▢ Yes, pharmacy **in** Tallaght used ▢ Yes, pharmacy **outside** of Tallaght used

▢ No, pharmacy not used ▢ Don't know

***If yes, pharmacy outside of Tallaght used skip to question 6.6***

***If no, pharmacy not used or don't know skip to question 6.7***

**6.5** Thinking of pharmacies in Tallaght used in the last 12 months complete the following:

***(Tick one on each line only)***

| How often did your household use pharmacies in Tallaght in the last 12 months? | ▢ Daily ▢ Weekly ▢ Monthly  ▢ Bimonthly ▢ Once or twice |
| --- | --- |
| Are pharmacies in Tallaght an asset to the community? | ▢ Yes ▢ No |
| **If no,** please indicate **why not**? |  |

***(Tick one on each line only)***

| How often did your household use pharmacies in Tallaght in the last 12 months? | ▢ Daily ▢ Weekly ▢ Monthly  ▢ Bimonthly ▢ Once or twice |
| --- | --- |
| Are pharmacies in Tallaght an asset to the community? | ▢ Yes ▢ No |
| **If no,** please indicate **why not**? |  |

**6.6** Why did your household use a pharmacy **outside** of Tallaght? __________________________________

________________________________________________________________________

**Dental Care**

**6.7** Did anyone in your household (including you) use a dentist in the last 12 months?

▢ Yes, dentist **in** Tallaght used ▢ Yes, dentist **outside** of Tallaght used

▢ No, dentist not used ▢ Don't know

***If yes, dentist outside of Tallaght used skip to question 6.9***

***If no dentist not used or don't know skip to question 6.10***

**6.8** Thinking of dentists in Tallaght used in the last 12 months complete the following:

***(Tick one on each line only)***

| How often did your household use dentists in Tallaght in the last 12 months? | ▢ Daily ▢ Weekly ▢ Monthly  ▢ Bimonthly ▢ Once or twice |
| --- | --- |
| Are dentists in Tallaght an asset to the community? | ▢ Yes ▢ No |
| **If no,** please indicate **why not**? |  |

***(Tick one on each line only)***

| How often did your household use dentists in Tallaght in the last 12 months? | ▢ Daily ▢ Weekly ▢ Monthly  ▢ Bimonthly ▢ Once or twice |
| --- | --- |
| Are dentists in Tallaght an asset to the community? | ▢ Yes ▢ No |
| **If no,** please indicate **why not**? |  |

**6.9** Why did your household use a dentist **outside** of Tallaght? ____________________________________

_______________________________________________________________________

| **Frequency Last 12 Months** |
| --- |
| **Daily:** 4 to 7 times per week **Weekly:** 1 to 3 times per week **Monthly:** 7 to 12 times a year  **Bimonthly:** 3 to 6 times a year **Once or twice:** 1 or 2 times a year |

**Other Healthcare Services**

**6.10** Did anyone in your household (including you) use other healthcare services in the last 12 months?

▢ Yes, other healthcare services **in** Tallaght used ▢ Yes, other healthcare services **outside** of Tallaght used

▢ No, other healthcare services not used ▢ Don't know

***If yes, other healthcare services outside of Tallaght used skip to question 6.12***

***If no other healthcare services not used or don't know skip to question 6.13***

**6.11** Thinking of other healthcare services in Tallaght used in the last 12 months complete the following:

***(Tick one on each line only)***

| How often did your household use other healthcare services in Tallaght in the last 12 months? | ▢ Daily ▢ Weekly ▢ Monthly  ▢ Bimonthly ▢ Once or twice |
| --- | --- |
| Are other healthcare services in Tallaght an asset to the community? | ▢ Yes ▢ No |
| **If no,** please indicate **why not**? |  |

***(Tick one on each line only)***

| How often did your household use other healthcare services in Tallaght in the last 12 months? | ▢ Daily ▢ Weekly ▢ Monthly  ▢ Bimonthly ▢ Once or twice |
| --- | --- |
| Are other healthcare services in Tallaght an asset to the community? | ▢ Yes ▢ No |
| **If no,** please indicate **why not**? |  |

**6.12** Why did your household use a healthcare service **outside** of Tallaght? _____________________________

________________________________________________________________________

**Mental Health Services**

**6.13** Did anyone in your household (including you) use mental health services in the last 12 months?

▢ Yes, mental health services **in** Tallaght used ▢ Yes, mental health services **outside** of Tallaght used

▢ No, mental health services not used ▢ Don't know

***If yes, mental health services outside of Tallaght used skip to question 6.15***

***If no mental health services not used or don't know skip to question 6.16***

**6.14** Thinking of mental health services in Tallaght used in the last 12 months complete the following:

***(Tick one on each line only)***

| How often did your household use mental health services in Tallaght in the last 12 months? | ▢ Daily ▢ Weekly ▢ Monthly  ▢ Bimonthly ▢ Once or twice |
| --- | --- |
| Are mental health services in Tallaght an asset to the community? | ▢ Yes ▢ No |
| **If no,** please indicate **why not**? |  |

***(Tick one on each line only)***

| How often did your household use mental health services in Tallaght in the last 12 months? | ▢ Daily ▢ Weekly ▢ Monthly  ▢ Bimonthly ▢ Once or twice |
| --- | --- |
| Are mental health services in Tallaght an asset to the community? | ▢ Yes ▢ No |
| **If no,** please indicate **why not**? |  |

**6.15** Why did your household use mental health services **outside** of Tallaght? ____________________________

________________________________________________________________________

| **Frequency Last 12 Months** |
| --- |
| **Daily:** 4 to 7 times per week **Weekly:** 1 to 3 times per week **Monthly:** 7 to 12 times a year  **Bimonthly:** 3 to 6 times a year **Once or twice:** 1 or 2 times a year |

**Support Groups**

**6.16** Did anyone in your household (including you) use support groups in the last 12 months?

▢ Yes, support groups **in** Tallaght used ▢ Yes, support groups **outside** of Tallaght used

▢ No, support groups not used ▢ Don't know

***If yes, support groups outside of Tallaght used skip to question 6.18***

***If no support groups not used or don't know skip to question 6.19***

**6.17** Thinking of support groups in Tallaght used in the last 12 months complete the following:

***(Tick one on each line only)***

| How often did your household use support groups in Tallaght in the last 12 months? | ▢ Daily ▢ Weekly ▢ Monthly  ▢ Bimonthly ▢ Once or twice |
| --- | --- |
| Are support groups in Tallaght an asset to the community? | ▢ Yes ▢ No |
| **If no,** please indicate **why not**? |  |

***(Tick one on each line only)***

| How often did your household use support groups in Tallaght in the last 12 months? | ▢ Daily ▢ Weekly ▢ Monthly  ▢ Bimonthly ▢ Once or twice |
| --- | --- |
| Are support groups in Tallaght an asset to the community? | ▢ Yes ▢ No |
| **If no,** please indicate **why not**? |  |

**6.18** Why did your household use support groups **outside** of Tallaght? ________________________________

________________________________________________________________________

**Addiction Services**

**6.19** Did anyone in your household (including you) use addiction services in the last 12 months?

▢ Yes, addiction services **in** Tallaght used ▢ Yes, addiction services **outside** of Tallaght used

▢ No, addiction services not used ▢ Don't know

***If yes, addiction services outside of Tallaght used skip to question 6.21***

***If no addiction services not used or don't know skip to question 6.22***

**6.20** Thinking of addiction services in Tallaght used in the last 12 months complete the following:

***(Tick one on each line only)***

| How often did your household use addiction services in Tallaght in the last 12 months? | ▢ Daily ▢ Weekly ▢ Monthly  ▢ Bimonthly ▢ Once or twice |
| --- | --- |
| Are addiction services in Tallaght an asset to the community? | ▢ Yes ▢ No |
| **If no,** please indicate **why not**? |  |

***(Tick one on each line only)***

| How often did your household use addiction services in Tallaght in the last 12 months? | ▢ Daily ▢ Weekly ▢ Monthly  ▢ Bimonthly ▢ Once or twice |
| --- | --- |
| Are addiction services in Tallaght an asset to the community? | ▢ Yes ▢ No |
| **If no,** please indicate **why not**? |  |

**6.21** Why did your household use addiction services **outside** of Tallaght? ______________________________

________________________________________________________________________

| **Frequency Last 12 Months** |
| --- |
| **Daily:** 4 to 7 times per week **Weekly:** 1 to 3 times per week **Monthly:** 7 to 12 times a year  **Bimonthly:** 3 to 6 times a year **Once or twice:** 1 or 2 times a year |

**Disability Services**

**6.22** Did anyone in your household (including you) use disability services in the last 12 months?

▢ Yes, disability services **in** Tallaght used ▢ Yes, disability services **outside** of Tallaght used

▢ No, disability services not used ▢ No, disability services n/a ▢ Don't know

***If yes, disability services outside of Tallaght used skip to question 6.24***

***If no disability services not used or n/a or don't know skip to section 7***

**6.23** Thinking of disability services in Tallaght used in the last 12 months complete the following:

***(Tick one on each line only)***

| How often did your household use disability services in Tallaght in the last 12 months? | ▢ Daily ▢ Weekly ▢ Monthly  ▢ Bimonthly ▢ Once or twice |
| --- | --- |
| Are disability services in Tallaght an asset to the community? | ▢ Yes ▢ No |
| **If no,** please indicate **why not**? |  |

***(Tick one on each line only)***

| How often did your household use disability services in Tallaght in the last 12 months? | ▢ Daily ▢ Weekly ▢ Monthly  ▢ Bimonthly ▢ Once or twice |
| --- | --- |
| Are disability services in Tallaght an asset to the community? | ▢ Yes ▢ No |
| **If no,** please indicate **why not**? |  |

**6.24** Why did your household use a disability services **outside** of Tallaght? _____________________________

_______________________________________________________________________

**Section 7: Education Inventory**

| **Frequency Last 12 Months** |
| --- |
| **Daily:** 4 to 7 times per week **Weekly:** 1 to 3 times per week **Monthly:** 7 to 12 times a year  **Bimonthly:** 3 to 6 times a year **Once or twice:** 1 or 2 times a year |

***Use laminates provided***

**Crèches /Nursery Schools**

**7.1** Did anyone in your household (including you) use crèches/nursery schools in the last 12 months?

▢ Yes, crèches/nursery schools **in** Tallaght used ▢ Yes, crèches/nursery schools **outside** of Tallaght used

▢ No, crèches/nursery schools not used ▢ No, crèches/nursery schools n/a ▢ Don't know

***If yes, crèches/nursery schools outside of Tallaght used skip to question 7.3***

***If no crèches/nursery schools not used or n/a or don't know skip to question 7.4***

**7.2** Thinking of crèches/nursery schools in Tallaght used in the last 12 months complete the following:

***(Tick one on each line only)***

| How often did your household use crèches/nursery schools in Tallaght in the last 12 months? | ▢ Daily ▢ Weekly ▢ Monthly  ▢ Bimonthly ▢ Once or twice |
| --- | --- |
| Are crèches/nursery schools in Tallaght an asset to the community? | ▢ Yes ▢ No |
| **If no,** please indicate **why not**? |  |

***(Tick one on each line only)***

| How often did your household use crèches/nursery schools in Tallaght in the last 12 months? | ▢ Daily ▢ Weekly ▢ Monthly  ▢ Bimonthly ▢ Once or twice |
| --- | --- |
| Are crèches/nursery schools in Tallaght an asset to the community? | ▢ Yes ▢ No |
| **If no,** please indicate **why not**? |  |

**7.3** Why did your household use crèches/nursery schools **outside** of Tallaght? ___________________________

_______________________________________________________________________

**Primary Schools**

**7.4** Did anyone in your household (including you) use primary schools in the last 12 months?

▢ Yes, primary schools **in** Tallaght used ▢ Yes, primary schools **outside** of Tallaght used

▢ No, primary schools not used ▢ No, primary schools n/a ▢ Don't know

***If yes, primary schools outside of Tallaght used skip to question 7.6***

***If no primary schools not used or n/a or don't know skip to question 7.7***

**7.5** Thinking of primary schools in Tallaght used in the last 12 months complete the following:

***(Tick one on each line only)***

| How often did your household use primary schools in Tallaght in the last 12 months? | ▢ Daily ▢ Weekly ▢ Monthly  ▢ Bimonthly ▢ Once or twice |
| --- | --- |
| Are primary schools in Tallaght an asset to the community? | ▢ Yes ▢ No |
| **If no,** please indicate **why not**? |  |

***(Tick one on each line only)***

| How often did your household use primary schools in Tallaght in the last 12 months? | ▢ Daily ▢ Weekly ▢ Monthly  ▢ Bimonthly ▢ Once or twice |
| --- | --- |
| Are primary schools in Tallaght an asset to the community? | ▢ Yes ▢ No |
| **If no,** please indicate **why not**? |  |

**7.6** Why did your household use primary schools **outside** of Tallaght? ________________________________

________________________________________________________________________

| **Frequency Last 12 Months** |
| --- |
| **Daily:** 4 to 7 times per week **Weekly:** 1 to 3 times per week **Monthly:** 7 to 12 times a year  **Bimonthly:** 3 to 6 times a year **Once or twice:** 1 or 2 times a year |

**Secondary Schools**

**7.7** Did anyone in your household (including you) use secondary schools in the last 12 months?

▢ Yes, secondary schools **in** Tallaght used ▢ Yes, secondary schools **outside** of Tallaght used

▢ No, secondary schools not used ▢ No, secondary schools n/a ▢ Don't know

***If yes, secondary schools outside of Tallaght used skip to question 7.9***

***If no secondary schools not used or n/a or don't know skip to question 7.10***

**7.8** Thinking of secondary schools in Tallaght used in the last 12 months complete the following:

***(Tick one on each line only)***

| How often did your household use secondary schools in Tallaght in the last 12 months? | ▢ Daily ▢ Weekly ▢ Monthly  ▢ Bimonthly ▢ Once or twice |
| --- | --- |
| Are secondary schools in Tallaght an asset to the community? | ▢ Yes ▢ No |
| **If no,** please indicate **why not**? |  |

***(Tick one on each line only)***

| How often did your household use secondary schools in Tallaght in the last 12 months? | ▢ Daily ▢ Weekly ▢ Monthly  ▢ Bimonthly ▢ Once or twice |
| --- | --- |
| Are p secondary schools in Tallaght an asset to the community? | ▢ Yes ▢ No |
| **If no,** please indicate **why not**? |  |

**7.9** Why did your household use secondary schools **outside** of Tallaght? ______________________________

________________________________________________________________________

**Third Level Education**

**7.10** Did anyone in your household (including you) use third level education in the last 12 months?

▢ Yes, third level education **in** Tallaght used ▢ Yes, third level education **outside** of Tallaght used

▢ No, third level education not used ▢ Don't know

***If yes, third level education outside of Tallaght used skip to question 7.12***

***If no third level education*** ***not used or don't know skip to question 7.13***

**7.11** Thinking of third level education in Tallaght used in the last 12 months complete the following:

***(Tick one on each line only)***

| How often did your household use third level education in Tallaght in the last 12 months? | ▢ Daily ▢ Weekly ▢ Monthly  ▢ Bimonthly ▢ Once or twice |
| --- | --- |
| Are third level education in Tallaght an asset to the community? | ▢ Yes ▢ No |
| **If no,** please indicate **why not**? |  |

**7.12** Why did your household use third level education **outside** of Tallaght? _____________________________

________________________________________________________________________

| **Frequency Last 12 Months** |
| --- |
| **Daily:** 4 to 7 times per week **Weekly:** 1 to 3 times per week **Monthly:** 7 to 12 times a year  **Bimonthly:** 3 to 6 times a year **Once or twice:** 1 or 2 times a year |

**Adult Education**

**7.13** Did anyone in your household (including you) use adult education services in the last 12 months?

▢ Yes, adult education services **in** Tallaght used ▢ Yes, adult education services **outside** of Tallaght used

▢ No, adult education services not used ▢ Don't know

***If yes, adult education services outside of Tallaght used skip to question 7.15***

***If no adult education services not used or don't know skip to question 7.16***

**7.14** Thinking of adult education services in Tallaght used in the last 12 months complete the following:

***(Tick one on each line only)***

| How often did your household use adult education services in Tallaght in the last 12 months? | ▢ Daily ▢ Weekly ▢ Monthly  ▢ Bimonthly ▢ Once or twice |
| --- | --- |
| Are adult education services in Tallaght an asset to the community? | ▢ Yes ▢ No |
| **If no,** please indicate **why not**? |  |

***(Tick one on each line only)***

| How often did your household use adult education services in Tallaght in the last 12 months? | ▢ Daily ▢ Weekly ▢ Monthly  ▢ Bimonthly ▢ Once or twice |
| --- | --- |
| Are adult education services in Tallaght an asset to the community? | ▢ Yes ▢ No |
| **If no,** please indicate **why not**? |  |

**7.15** Why did your household use adult education services **outside** of Tallaght? __________________________

________________________________________________________________________

**Training and Employment Services**

**7.16** Did anyone in your household (including you) use training and employment services in the last 12 months?

▢ Yes, training and employment services **in** Tallaght used ▢ Yes, training and employment services **outside** of Tallaght used

▢ No, training and employment services not used ▢ Don't know

***If yes, training and employment services outside of Tallaght used skip to question 7.18***

***If no training and employment services not used or don't know skip to section 8***

**7.17** Thinking of training and employment services in Tallaght used in the last 12 months complete the following:

***(Tick one on each line only)***

| How often did your household use training and employment services in Tallaght in the last 12 months? | ▢ Daily ▢ Weekly ▢ Monthly  ▢ Bimonthly ▢ Once or twice |
| --- | --- |
| Are training and employment services in Tallaght an asset to the community? | ▢ Yes ▢ No |
| **If no,** please indicate **why not**? |  |

***(Tick one on each line only)***

| How often did your household use training and employment services in Tallaght in the last 12 months? | ▢ Daily ▢ Weekly ▢ Monthly  ▢ Bimonthly ▢ Once or twice |
| --- | --- |
| Are training and employment services in Tallaght an asset to the community? | ▢ Yes ▢ No |
| **If no,** please indicate **why not**? |  |

**7.18** Why did your household use training and employment services **outside** of Tallaght? _____________________

________________________________________________________________________

**Section 8: Community Facilities Inventory**

| **Frequency Last 12 Months** |
| --- |
| **Daily:** 4 to 7 times per week **Weekly:** 1 to 3 times per week **Monthly:** 7 to 12 times a year  **Bimonthly:** 3 to 6 times a year **Once or twice:** 1 or 2 times a year |

***Use laminates provided***

**Parks**

**8.1** Did anyone in your household (including you) use parks in the last 12 months?

▢ Yes, parks **in** Tallaght used ▢ Yes, parks **outside** of Tallaght used

▢ No, parks not used ▢ Don't know

***If yes, parks outside of Tallaght used skip to question 8.3***

***If no parks not used or don't know skip to question 8.4***

**8.2** Thinking of parks in Tallaght used in the last 12 months complete the following:

***(Tick one on each line only)***

| How often did your household use parks in Tallaght in the last 12 months? | ▢ Daily ▢ Weekly ▢ Monthly  ▢ Bimonthly ▢ Once or twice |
| --- | --- |
| Are parks in Tallaght an asset to the community? | ▢ Yes ▢ No |
| **If no,** please indicate **why not**? |  |

***(Tick one on each line only)***

| How often did your household use parks in Tallaght in the last 12 months? | ▢ Daily ▢ Weekly ▢ Monthly  ▢ Bimonthly ▢ Once or twice |
| --- | --- |
| Are parks in Tallaght an asset to the community? | ▢ Yes ▢ No |
| **If no,** please indicate **why not**? |  |

**8.3** Why did your household use parks **outside** of Tallaght? ______________________________________

________________________________________________________________________

**Playgrounds**

**8.4** Did anyone in your household (including you) use playgrounds in the last 12 months?

▢ Yes, playgrounds **in** Tallaght used ▢ Yes, parks **outside** of Tallaght used

▢ No, playgrounds not used ▢ Don't know

***If yes, playgrounds outside of Tallaght used skip to question 8.6***

***If no playgrounds not used or don't know skip to question 8.7***

**8.5** Thinking of playgrounds in Tallaght used in the last 12 months complete the following:

***(Tick one on each line only)***

| How often did your household use playgrounds in Tallaght in the last 12 months? | ▢ Daily ▢ Weekly ▢ Monthly  ▢ Bimonthly ▢ Once or twice |
| --- | --- |
| Are playgrounds in Tallaght an asset to the community? | ▢ Yes ▢ No |
| **If no,** please indicate **why not**? |  |

***(Tick one on each line only)***

| How often did your household use playgrounds in Tallaght in the last 12 months? | ▢ Daily ▢ Weekly ▢ Monthly  ▢ Bimonthly ▢ Once or twice |
| --- | --- |
| Are playgrounds in Tallaght an asset to the community? | ▢ Yes ▢ No |
| **If no,** please indicate **why not**? |  |

**8.6** Why did your household use playgrounds **outside** of Tallaght? __________________________________

____________________________________________________________________________________

| **Frequency Last 12 Months** |
| --- |
| **Daily:** 4 to 7 times per week **Weekly:** 1 to 3 times per week **Monthly:** 7 to 12 times a year  **Bimonthly:** 3 to 6 times a year **Once or twice:** 1 or 2 times a year |

**Community Centres**

**8.7** Did anyone in your household (including you) use community centres in the last 12 months?

▢ Yes, community centres **in** Tallaght used ▢ Yes, community centres **outside** of Tallaght used

▢ No, community centres not used ▢ Don't know

***If yes, community centres outside of Tallaght used skip to question 8.9***

***If no community centres not used or don't know skip to question 8.10***

**8.8** Thinking of community centres in Tallaght used in the last 12 months complete the following:

***(Tick one on each line only)***

| How often did your household use community centres in Tallaght in the last 12 months? | ▢ Daily ▢ Weekly ▢ Monthly  ▢ Bimonthly ▢ Once or twice |
| --- | --- |
| Are community centres in Tallaght an asset to the community? | ▢ Yes ▢ No |
| **If no,** please indicate **why not**? |  |

***(Tick one on each line only)***

| How often did your household use community centres in Tallaght in the last 12 months? | ▢ Daily ▢ Weekly ▢ Monthly  ▢ Bimonthly ▢ Once or twice |
| --- | --- |
| Are community centres in Tallaght an asset to the community? | ▢ Yes ▢ No |
| **If no,** please indicate **why not**? |  |

**8.9** Why did your household use community centres **outside** of Tallaght? ______________________________

________________________________________________________________________

**Community Services**

**8.10** Did anyone in your household (including you) use community services in the last 12 months?

▢ Yes, community services **in** Tallaght used ▢ Yes, community services **outside** of Tallaght used

▢ No, community services not used ▢ Don't know

***If yes, community services outside of Tallaght used skip to question 8.12***

***If no community services not used or don't know skip to question 8.13***

**8.11** Thinking of community services in Tallaght used in the last 12 months complete the following:

***(Tick one on each line only)***

| How often did your household use community services in Tallaght in the last 12 months? | ▢ Daily ▢ Weekly ▢ Monthly  ▢ Bimonthly ▢ Once or twice |
| --- | --- |
| Are community services in Tallaght an asset to the community? | ▢ Yes ▢ No |
| **If no,** please indicate **why not**? |  |

***(Tick one on each line only)***

| How often did your household use community services in Tallaght in the last 12 months? | ▢ Daily ▢ Weekly ▢ Monthly  ▢ Bimonthly ▢ Once or twice |
| --- | --- |
| Are community services in Tallaght an asset to the community? | ▢ Yes ▢ No |
| **If no,** please indicate **why not**? |  |

**8.12** Why did your household use community services **outside** of Tallaght? _____________________________

________________________________________________________________________

| **Frequency Last 12 Months** |
| --- |
| **Daily:** 4 to 7 times per week **Weekly:** 1 to 3 times per week **Monthly:** 7 to 12 times a year  **Bimonthly:** 3 to 6 times a year **Once or twice:** 1 or 2 times a year |

**Churches/Places of Worship**

**8.13** Did anyone in your household (including you) use churches/places of worship in the last 12 months?

▢ Yes, churches/places of worship **in** Tallaght used ▢ Yes, churches/places of worship **outside** of Tallaght used

▢ No, churches/places of worship not used ▢ Don't know

***If yes, churches/places of worship*** ***outside of Tallaght used skip to question 8.15***

***If no churches/places of worship not used or don't know skip to question 8.16***

**8.14** Thinking of churches/places of worship in Tallaght used in the last 12 months complete the following:

***(Tick one on each line only)***

| How often did your household use churches/places of worship in Tallaght in the last 12 months? | ▢ Daily ▢ Weekly ▢ Monthly  ▢ Bimonthly ▢ Once or twice |
| --- | --- |
| Are churches/places of worship in Tallaght an asset to the community? | ▢ Yes ▢ No |
| **If no,** please indicate **why not**? |  |

***(Tick one on each line only)***

| How often did your household use churches/places of worship in Tallaght in the last 12 months? | ▢ Daily ▢ Weekly ▢ Monthly  ▢ Bimonthly ▢ Once or twice |
| --- | --- |
| Are churches/places of worship in Tallaght an asset to the community? | ▢ Yes ▢ No |
| **If no,** please indicate **why not**? |  |

**8.15** Why did your household use churches/places of worship **outside** of Tallaght? _________________________

________________________________________________________________________

**Youth Services**

**8.16** Did anyone in your household (including you) use youth services in the last 12 months?

▢ Yes, youth services **in** Tallaght used ▢ Yes, youth services **outside** of Tallaght used

▢ No, youth services not used ▢ No, youth services n/a ▢ Don't know

***If yes, youth services outside of Tallaght used skip to question 8.18***

***If no youth services not used or n/a or don't know skip to question 8.19***

**8.17** Thinking of youth services in Tallaght used in the last 12 months complete the following:

***(Tick one on each line only)***

| How often did your household use youth services in Tallaght in the last 12 months? | ▢ Daily ▢ Weekly ▢ Monthly  ▢ Bimonthly ▢ Once or twice |
| --- | --- |
| Are youth services in Tallaght an asset to the community? | ▢ Yes ▢ No |
| **If no,** please indicate **why not**? |  |

***(Tick one on each line only)***

| How often did your household use youth services in Tallaght in the last 12 months? | ▢ Daily ▢ Weekly ▢ Monthly  ▢ Bimonthly ▢ Once or twice |
| --- | --- |
| Are youth services in Tallaght an asset to the community? | ▢ Yes ▢ No |
| **If no,** please indicate **why not**? |  |

**8.18** Why did your household use youth services **outside** of Tallaght? ________________________________

________________________________________________________________________

| **Last 12 Months Frequency Response Card** |
| --- |
| **Daily:** 4 to 7 times per week **Weekly:** 1 to 3 times per week **Monthly:** 7 to 12 times  **Bimonthly:** 3 to 6 times **Once or twice:** 1 or 2 times |

**Senior Citizen Services**

**8.19** Did anyone in your household (including you) use senior citizen services in the last 12 months?

▢ Yes, senior citizen services **in** Tallaght used ▢ Yes, senior citizen services **outside** of Tallaght used

▢ No, senior citizen services not used ▢ No, senior citizen services n/a ▢ Don't know

***If yes, senior citizen services outside of Tallaght used skip to question 8.21***

***If no senior citizen services not used or n/a or don't know skip to question 8.22***

**8.20** Thinking of senior citizen services in Tallaght used in the last 12 months complete the following:

***(Tick one on each line only)***

| How often did your household use senior citizen services in Tallaght in the last 12 months? | ▢ Daily ▢ Weekly ▢ Monthly  ▢ Bimonthly ▢ Once or twice |
| --- | --- |
| Are senior citizen services in Tallaght an asset to the community? | ▢ Yes ▢ No |
| **If no,** please indicate **why not**? |  |

***(Tick one on each line only)***

| How often did your household use senior citizen services in Tallaght in the last 12 months? | ▢ Daily ▢ Weekly ▢ Monthly  ▢ Bimonthly ▢ Once or twice |
| --- | --- |
| Are senior citizen services in Tallaght an asset to the community? | ▢ Yes ▢ No |
| **If no,** please indicate **why not**? |  |

**8.21** Why did your household use senior citizen services **outside** of Tallaght? ___________________________

________________________________________________________________________

**Transport Services**

**8.22** Did anyone in your household (including you) use transport services in the last 12 months?

▢ Yes, transport services **in** Tallaght used ▢ Yes, transport services **outside** of Tallaght used

▢ No, transport services not used ▢ Don't know

***If yes, transport services outside of Tallaght used skip to question 8.24***

***If no transport services not used or don't know skip to question 8.25***

**8.23** Thinking of transport services in Tallaght used in the last 12 months complete the following:

***(Tick one on each line only)***

| How often did your household use transport services in Tallaght in the last 12 months? | ▢ Daily ▢ Weekly ▢ Monthly  ▢ Bimonthly ▢ Once or twice |
| --- | --- |
| Are transport services in Tallaght an asset to the community? | ▢ Yes ▢ No |
| **If no,** please indicate **why not**? |  |

***(Tick one on each line only)***

| How often did your household use transport services in Tallaght in the last 12 months? | ▢ Daily ▢ Weekly ▢ Monthly  ▢ Bimonthly ▢ Once or twice |
| --- | --- |
| Are transport services in Tallaght an asset to the community? | ▢ Yes ▢ No |
| **If no,** please indicate **why not**? |  |

**8.24** Why did your household use transport services **outside** of Tallaght? ______________________________

________________________________________________________________________

| **Frequency Last 12 Months** |
| --- |
| **Daily:** 4 to 7 times per week **Weekly:** 1 to 3 times per week **Monthly:** 7 to 12 times a year  **Bimonthly:** 3 to 6 times a year **Once or twice:** 1 or 2 times a year |

**Hobby Facilities**

**8.25** Did anyone in your household (including you) use hobby facilities in the last 12 months?

▢ Yes, hobby facilities **in** Tallaght used ▢ Yes, hobby facilities **outside** of Tallaght used

▢ No, hobby facilities not used ▢ Don't know

***If yes, hobby facilities outside of Tallaght used skip to question 8.27***

***If no hobby facilities not used or don't know skip to question 8.28***

**8.26** Thinking of hobby facilities in Tallaght used in the last 12 months complete the following:

***(Tick one on each line only)***

| How often did your household use hobby facilities in Tallaght in the last 12 months? | ▢ Daily ▢ Weekly ▢ Monthly  ▢ Bimonthly ▢ Once or twice |
| --- | --- |
| Are hobby facilities in Tallaght an asset to the community? | ▢ Yes ▢ No |
| **If no,** please indicate **why not**? |  |

***(Tick one on each line only)***

| How often did your household use hobby facilities in Tallaght in the last 12 months? | ▢ Daily ▢ Weekly ▢ Monthly  ▢ Bimonthly ▢ Once or twice |
| --- | --- |
| Are hobby facilities in Tallaght an asset to the community? | ▢ Yes ▢ No |
| **If no,** please indicate **why not**? |  |

**8.27** Why did your household use hobby facilities **outside** of Tallaght? _______________________________

________________________________________________________________________

**Other Services**

**8.28** Did anyone in your household (including you) use other services in the last 12 months?

▢ Yes, other services **in** Tallaght used ▢ Yes, other services **outside** of Tallaght used

▢ No, other services not used ▢ Don't know

***If yes, other services outside of Tallaght used skip to question 8.30***

***If no other services not used or don't know skip to section 9***

**8.29** Thinking of other services in Tallaght used in the last 12 months complete the following:

***(Tick one on each line only)***

| How often did your household use other services in Tallaght in the last 12 months? | ▢ Daily ▢ Weekly ▢ Monthly  ▢ Bimonthly ▢ Once or twice |
| --- | --- |
| Are other services in Tallaght an asset to the community? | ▢ Yes ▢ No |
| **If no,** please indicate **why not**? |  |

***(Tick one on each line only)***

| How often did your household use other services in Tallaght in the last 12 months? | ▢ Daily ▢ Weekly ▢ Monthly  ▢ Bimonthly ▢ Once or twice |
| --- | --- |
| Are other services in Tallaght an asset to the community? | ▢ Yes ▢ No |
| **If no,** please indicate **why not**? |  |

**8.30** Why did your household use other services **outside** of Tallaght? ________________________________

________________________________________________________________________

**Section 9: Sport and Hobby Facility Inventory**

| **Frequency Last 12 Months** |
| --- |
| **Daily:** 4 to 7 times per week **Weekly:** 1 to 3 times per week **Monthly:** 7 to 12 times a year  **Bimonthly:** 3 to 6 times a year **Once or twice:** 1 or 2 times a year |

***Use laminates provided***

**Sports Clubs and Facilities**

**9.1** Did anyone in your household (including you) use sports clubs or facilities in the last 12 months?

▢ Yes, sports clubs/facilities **in** Tallaght used ▢ Yes, sports clubs/facilities **outside** of Tallaght used

▢ No, sports clubs not used ▢ Don't know

***If yes, sports club/facilities outside of Tallaght used skip to question 9.3***

***If no, sports clubs/facilities not used or don't know skip to section 10***

**9.2** Thinking of sports clubs and facilities in Tallaght used in the last 12 months complete the following:

***(Tick one on each line only)***

| How often did your household use sports clubs and facilities in Tallaght in the last 12 months? | ▢ Daily ▢ Weekly ▢ Monthly  ▢ Bimonthly ▢ Once or twice |
| --- | --- |
| Are sports clubs and facilities in Tallaght an asset to the community? | ▢ Yes ▢ No |
| **If no,** please indicate **why not**? |  |

***(Tick one on each line only)***

| How often did your household use sports clubs and facilities in Tallaght in the last 12 months? | ▢ Daily ▢ Weekly ▢ Monthly  ▢ Bimonthly ▢ Once or twice |
| --- | --- |
| Are sports clubs and facilities in Tallaght an asset to the community? | ▢ Yes ▢ No |
| **If no,** please indicate **why not**? |  |

***(Tick one on each line only)***

| How often did your household use sports clubs and facilities in Tallaght in the last 12 months? | ▢ Daily ▢ Weekly ▢ Monthly  ▢ Bimonthly ▢ Once or twice |
| --- | --- |
| Are sports clubs and facilities in Tallaght an asset to the community? | ▢ Yes ▢ No |
| **If no,** please indicate **why not**? |  |

***(Tick one on each line only)***

| How often did your household use sports clubs and facilities in Tallaght in the last 12 months? | ▢ Daily ▢ Weekly ▢ Monthly  ▢ Bimonthly ▢ Once or twice |
| --- | --- |
| Are sports clubs and facilities in Tallaght an asset to the community? | ▢ Yes ▢ No |
| **If no,** please indicate **why not**? |  |

**9.3** Why did your household use sports clubs/facilities **outside** of Tallaght? ________________________________________

___________________________________________________________________________________________________

**Section 10: Missing Assets**

**10.1** Is there anything missing from this list which is an asset to your life in Tallaght?

________________________________________________________________________

________________________________________________________________________

________________________________________________________________________

**End of questionnaire**

**Thank you**
